# Cilia, *tubby* mice, and obesity

**DOI:** 10.1186/2046-2530-2-1

**Published:** 2013-01-03

**Authors:** Saikat Mukhopadhyay, Peter K Jackson

**Affiliations:** 1 Department of Research Oncology, Genentech Inc, South San Francisco, CA, 94080, USA; 2 Department of Cell Biology, UT Southwestern Medical Center, 5323 Harry Hines Boulevard, Dallas, TX 75390, USA

## Abstract

Primary cilia have been previously linked to the central regulation of satiety. The *tubby* mouse is characterized by maturity-onset obesity and blindness. A recent paper demonstrates molecular defects in trafficking of ciliary GPCRs in the central neurons of *tubby* mice, underscoring the role of ciliary signaling in the pathogenesis of this monogenic obesity syndrome.

Please see related Research article by Li et al., http://www.ciliajournal.com/content/1/1/21

## Background

Most neurons in the vertebrate nervous system elaborate primary cilia. Historically, neuronal primary cilia were first identified in neuroepithelial progenitor cells projecting into the neural tube lumen. Later on, they were described to be broadly present both in neurons and glia [1]. The primary cilia function as sensory antennae in a wide variety of cells. Cilia-localized receptors, which include certain G protein-coupled receptors (GPCRs), and their downstream effectors, determine the sensory modality of cells in specific contexts, especially during vertebrate photoreception or olfaction and for responding to morphogens, such as Sonic hedgehog (Shh). Although we have come to appreciate the function of the primary cilium in other tissues and organisms, the functional roles of this ubiquitous neuronal organelle in integrating neuroendocrine signals have remained enigmatic. Diseases resulting from disruption of primary cilia and the associated basal body complex, called ciliopathies, often have strong neurological components, emphasizing the role of this cellular compartment in neural development [2]. Interestingly, aside from the strong neurodevelopmental phenotypes, progressive obesity often affects patients with certain ciliopathies such as the Bardet-Biedel Syndrome (BBS) and Alström Syndrome [3]. Notably, conditional knockout of components of the cilia in the mice hypothalamus results in hyperphagia-induced obesity and underscores the role of ciliary signaling in the central regulation of satiety [4]. Thus, it is imperative to achieve a better understanding of the ciliary signaling pathways in central satiety networks, which could lead to novel ways for treating the global obesity pandemic.

The *tubby* mouse was initially identified as a spontaneous maturity-onset obesity syndrome [5], and positional cloning strategies in the 1990s mapped the causative mutation to a novel gene of unknown function called *Tub*[6,7]. In nematodes, *tub-1*, the canonical *Tub* homolog was identified in an RNAi screen for fat storage defects [8], and was found to be expressed in the ciliated neurons [9], highlighting the role of neuroendocrine signals in maintenance of systemic fat homeostasis even in these distant evolutionary relatives. Thanks to a recent paper from Sun *et al*. [10] the *tubby* mouse can now be added to the growing list of monogenic obesity syndromes with a strong ciliary functional component in the central nervous system [3]. The authors demonstrate molecular defects in ciliary GPCR signaling in the *tubby* mice, suggesting the importance of ciliary GPCR trafficking in central neurons implicated in satiety circuits.

The authors show that in the *tubby* mice, the primary cilia in the neurons show no obvious structural defects. However, two ciliary GPCRs, melanin-concentrating hormone receptor 1 (Mchr1) and somatostatin receptor subtype 3 (Sstr3), known to localize to distinct regions of the brain [11,12], are strongly prevented from trafficking to the primary cilia. This phenotype is strongly reminiscent of a previous study showing defective ciliary targeting of these receptors in BBS mice [12]. Similar to the BBS mice, the *tubby* mice also display retinal degeneration, and a defect in trafficking of rhodopsin to the outer segment of the photoreceptor, an extension of the connecting cilia in these cells. However, in distinction from the BBS mice that have defective olfactory cilia and are anosmic [13], *tubby* mice do not show defects in either the structure of these specialized cilia or in localization of olfactory GPCRs. This difference could be because *Tub* is not expressed and does not play a major role in these specific neurons, or because the presence of other tubby family homologs (such as *Tulp3*) compensates for the loss of Tub activity. The authors also detect the defects in ciliary GPCR trafficking well in advance of the development of obesity and retinal degeneration, implying that these trafficking defects could be causative for the development of these phenotypes.

How does Tubby affect ciliary GPCR trafficking? The *Tub* gene is the founding member of a family of proteins [14], characterized by a C-terminal tubby domain, which is highly specific for binding to 4,5 phosphoinsositides (PIP_2_) [15]. This domain is likely to participate in binding to specific membrane compartments, which for Tubby may be the ciliary membrane. Some of the tubby family members (including Tub, Tulp2 and Tulp3) also have a signature motif in the divergent N-terminus that binds to the core subunits of the ciliary intraflagellar transport complex-A (IFT-A) [16]. *Tulp3* mutant mice are embryonic lethal by mid-gestation [17], but previous *in vitro* studies with heterologous cultured ciliated cell lines suggested that both Tulp3 and IFT-A core subcomplex direct GPCR trafficking to the cilia [16]. Careful mutational analysis of both the IFT-A binding N-terminal and PIP_2_-binding C-terminal domains suggest that both the IFT-A- and membrane phosphoinositide-binding properties of TULP3 are necessary for ciliary GPCR localization. TULP3 thus bridges the IFT-A complex to the membrane compartment in gating ciliary GPCR trafficking, although the specific mechanism of ciliary GPCR recruitment remains to be determined. Most importantly, in the context of neuronal ciliary GPCR trafficking, the Tulp3 N-terminal fragment can act as a dominant negative reagent, preventing GPCR trafficking in cultured hippocampal neurons [16]. Tub also shares the IFT-A binding motif with Tulp3, and binds to the IFT-A complex [16], although possibly less efficiently. Thus, similar to Tulp3, Tub could be directing ciliary GPCR trafficking through its simultaneous binding to the IFT-A complex, and membrane phosphoinositides. Presumably, higher levels of Tub in the brain could compensate for the lower binding or weaker affinity of Tub for the IFT-A complex. According to the Allen Brain Atlas, hypothalamic *Tub* transcript levels are about 26 times that of *Tulp3*. However, Tub/IFT-A might also require additional factors. Besides, the dominant negative IFT-A-binding N-terminal fragment of Tulp3 would be expected to inhibit both Tulp3 and Tub binding to the IFT-A complex in these neurons, effectively shutting down complementary effects of these proteins in trafficking ciliary GPCRs. Thus, based on the spatial and temporal expression of these specific tubby family proteins in different tissues, and their affinity for the IFT-A complex, we might expect to observe a differential effect in their relative capacities for gating ciliary GPCRs. These differences could create a combinatorial code by utilizing an identical molecular mechanism for fine-tuning levels of ciliary receptors.

A suggestion implicit in the authors’ findings is that the GPCR trafficking defects into the neuronal cilia, especially Mchr1, could underlie the obesity phenotype in the *tubby* mice. Mchr1, the receptor for melanin-concentrating hormone (MCH), is involved in the regulation of feeding and energy balance [18,19]. However, *Mchr1* knockout mice are lean [18,19], whereas MCH overexpression results in obesity [20]. Thus, in the simplest model, Mchr1 trafficking defect to the cilia should mirror its effect on energy balance and cause leanness, rather than obesity, as evident in the *tubby* mouse. Dissecting the downstream effectors of Mchr1 in regulating energy balance could address the conflicting effects of Mchr1 trafficking on obesity. The best downstream effector implicated in neuronal satiety pathways is the adenylyl cyclase, type 3 (ACIII). Mice deficient in ACIII become obese with age, suggesting that ACIII-mediated cAMP signals are critical in the hypothalamus [21]. In line with this observation, downstream effectors of MCHR1 signaling include multiple G proteins including G_i_, G_o_ and G_q_[22]. Thus, MCHR1 inhibits cAMP production stimulated by forskolin and increases intracellular Ca^2+^ levels. However, in metabolically active brain slices, it paradoxically increases extracellular signal-regulated kinase (ERK) phosphorylation to levels above those observed with forskolin alone [23]. Thus, the synergistic effects of Mchr1 signaling on cAMP, Ca^2+^, and ERK phosphorylation could be important in determining the final outcome on promoting energy intake.

Another possibility is the role of additional ciliary GPCRs in neuronal satiety centers, and a combination of trafficking defects of these receptors could result in the final maturity-onset obesity phenotype. For example, other neuronal GPCRs such as D1, D2, and D5 dopamine receptors are also expressed in neuronal cilia [24], and were not examined in this study. Besides, it is important to note that our catalog of GPCRs expressed in neuronal cilia is mostly incomplete. Thus, although the exact molecular explanation for obesity in the *tubby* (and BBS) mice is far from clear, we still favor the hypothesis that mislocalization of other novel, yet-unidentified GPCRs could provide us with a more complete answer in the future. Nevertheless, the final acid test for dissecting the role of ciliary trafficking of these individual receptors on neuronal phenotypes would entail detailed engineering of *knock-in* mice, expressing ciliary localization-defective variants into the endogenous genomic loci of these receptors.

Another central question is the means by which ciliary signaling impacts neurons, and the reason why neurons need this signaling organelle in the first place. Currently, this is best answered in the case of morphogenetic developmental processes involving Shh signaling, which impacts neuronal differentiation both during embryogenesis and later stages. For example, Shh signaling in the cilia is fundamentally important in the neural progenitor cells during neural tube patterning [25]. Many of the Shh signaling components are localized to the cilia-basal body complex, and downstream signaling mediated by protein kinase A (PKA) and Gli3 processing are intricately linked to this organelle. In a broader developmental context, primary cilia are also fundamentally important in neurogenesis in cerebellar granule neurons [26,27], hippocampal neurogenesis in the dentate gyrus (DG) [28,29], adult DG neural stem cells [30], and in cerebral cortical development [31,32]. At least, in some of these neuronal cells, the primary cilium probably acts as a subcellular compartment for efficiently amplifying extracellular Shh signals for intracellular signal transduction. However, neither *tubby* nor BBS mice demonstrate gross defects in the neuroanatomical networks that regulate satiety, suggesting by extension that a lack of GPCR trafficking in these neurons probably would not cause apparent deficits during development of these circuits.

Apart from the role of cilia in Shh signaling and differentiation, recent studies have begun to provide intriguing molecular insights into other neuron-dependent processes dependent on the presence of cilia, and similar mechanisms could impact the satiety networks in a cilia-dependent manner. First of all, primary cilia function in glutamatergic synaptic integration of adult-born neurons [33]. Conditional deletion of cilia from adult-born neurons induces severe defects in dendritic refinement and synapse formation, which is partially correlated with an enhancement of Wnt and β-catenin signaling [33]. Signaling in the context of primary cilia could thus eventually impinge upon the subsequent efficient integration of neurons into neural networks. Second, primary ciliary signaling has also been shown to have an effect on long-term potentiation (LTP) and plasticity [34]. Sstr3 signaling in the hippocampus is important in novelty detection in mice, and adenylyl cyclase/cAMP-mediated LTP is impaired in hippocampal slices from the *Sstr3* knockout and upon addition of Sstr3 antagonists into wild-type sections. In this case, cilia could act as coincidence detectors and affect synaptic plasticity by affecting downstream signaling pathways. On a similar note, dopamine produces a synapse-specific enhancement of early LTP through D1/D5 receptors and cAMP signaling [35]. Future work is needed to establish if efficient targeting of these receptors to the neuronal cilia is important in these processes. Identifying downstream pathways regulating synaptic plasticity particularly promises to be an important future avenue of research for understanding the puzzling role of cilia in neuronal function.

## Competing interest

The authors declare that they have no competing interests.

## Authors’ contribution

All authors read and approved the final manuscript.
